# Bioactive-glass in Endodontic Therapy and Associated Microsurgery

**DOI:** 10.2174/1874210601711010164

**Published:** 2017-03-31

**Authors:** Andrea Corrado Profeta, Gian Marco Prucher

**Affiliations:** International Dental Clinic IDC, Casablanca, Morocco

**Keywords:** Bioactive-glass, Root canal treatment, Endodontic surgery, Root-end filling, Bone grafting

## Abstract

**Introduction:**

Bioactive-glass (B-G) has become a valuable adjunct to promote hard-tissue healing in many clinical situations and is of particular interest for endodontic care because of its biocompatibility, regenerative and antimicrobial properties as well as chemical composition that closely resembles the mineral make-up of human bone and dentine.

**Therapy:**

Initial studies suggested that bacteria-tight sealing within the entire root canal system can be achieved and successfully maintained after orthograde treatment. Promising results have also been obtained in conjunction with microsurgical techniques, with the aim of enhancing wound healing and positively influencing bone regeneration.

**Conclusion:**

Here, relevant literature was explored to present a comprehensive review of the rationale, development, and current applications of B-G in Endodontology illustrating them with case reports.

## INTRODUCTION

The utilization of biomaterials is an established feature in Dentistry and their importance is readily acknowledged. Research in Endodontology often involves proven materials that have been thoroughly tested by scientific investigation and clinical usage, as well as others that are the result of new knowledge and expectation to improve treatment outcomes in terms of percentages of success. Bioactive-glass (B-G, henceforth used in the text for brevity) has become a valuable adjunct to promote hard-tissue healing in many situations [1-3] and is of particular interest for endodontic care because its chemical composition (silicium, sodium, calcium and phosphorus oxides with specific weight percentages) closely resembles the mineral make-up of human bone and dentine. Considering the excellent biocompatibility, regenerative and antimicrobial properties of this material and the fact that it has been in use for more than forty years [4], its range of dental applications is still small. The goal of this investigation was to evaluate the use of B-G in endodontic therapy and associated microsurgery, and provide clinical findings. Clinical records and radiographic images were collected from patients who had undergone root canal treatment or periradicular surgery in private practice and had a minimum 1-year follow-up, which was deemed sufficient in predicting healing after surgery [5]. As part of the review process, original articles published in English from 1971 (year of B-G being introduced) [4] up to, and including, 21^st^ August 2016 were considered for the discussion of its advantages in terms of efficacy and efficiency. The search of bibliographical databases including medline (pubmed), web of science, scopus, scielo and cochrane was performed by using keywords as well as MeSH terms in addition to selective hand-searching of citations contained within located articles. The biochemical and cellular mechanisms of action and the role that they play in the healing process were also elucidated.

## ORTHOGRADE ROOT CANAL THERAPY

Obturation materials should form a bacteria-tight seal within the entire root canal system [6]. Therefore, they must ensure impervious sealing throughout long periods and should be volumetrically stable (or expand slightly). Gutta-percha, introduced as exclusive material in endodontic therapy [7], is thermoplastic and hence can be warmed to improve its adaptation to the complex root canal anatomy. It must be emphasized that heated gutta-percha shrinks considerably upon cooling to body temperature [8]. To compensate for this shrinkage, elaborate application techniques and a canal wall lining material, that is a sealer, have been adopted [9]. Sealers are not necessarily bioinert when extruded beyond the apex and in contact with periapical bone [10]. Furthermore, sealers may adhere with varying strength to gutta-percha and the root canal wall, resulting in interfacial gaps, microchannels, porosities and thus coronal-apical leakage. Calcium silicate-based cements have gained wide acceptance in the endodontic community to fill teeth with open apices, for perforation repair and as root-end filling materials, owing to their good physico-chemical and biological properties [11]. It is known that they adhere to hydrated dentinal walls by forming a crystalline bond *via* a diffusion-controlled process, a feature also reported for a restricted group of synthetic, commercially available bone substitutes, usually surface reactive glass-ceramics. Among these is B-G (grade 45S5, US Biomaterials Co., Alachua, FL, USA), the first man-made material to bond to bone [4], that is based on a simple four-component system of minerals normally found in the skeleton and teeth such as silica [SiO_2_ (46.1 wt.%)], sodium oxide [Na_2_O (24.4 wt.%)], calcium oxide [CaO (26.9 wt.%)], and phosphorus pentoxide [P_2_O_5_ (2.6 wt.%)]. B-G has been successfully used in dentine remineralization procedures [12] due to its ability to dissolve, upon contact with physiological body fluids or human plasma, and promote mineral precipitation with subsequent crystallization of hydroxyl carbonate apatite (HCA) on the glass/tissue interface. The detailed analysis of the reactions involved has been presented by Hench [4]. The process involves five stages which occur very rapidly on the surface of B-G particles because of fast ion exchange of alkali ions with hydrogen ions from the liquid medium (stage 1), glass network dissolution (stage 2), condensation of a silica-rich mass (stage 3), and calcium phosphate precipitation followed by crystallisation of the HCA layer within hours (stages 4 and 5). Such a phase is chemically and structurally similar to the mineral phase of human bone and dentine, allowing accelerated interfacial fusion and consolidation without toxicological consequences [12]. Unfortunately, root canals filled solely with a calcium silicate-based cement can never be retreated, a corrective measure that is deemed important by most clinicians. For this reason it was suggested to incorporate crystallizing particles into matrix polymers of core root filling materials. The premise was that the formation of calcium phosphate precipitates on the material’s surface under moist conditions, once accomplished, should render the conventional restoration more adhesive in root canals, which are inherently wet [7, 13]. In an initial study, incorporation of B-G into polyisoprene, the matrix polymer of gutta-percha, proved to enhance the dentinal sealing due to the compound’s hydrophilic properties and the resultant moisture expansion towards the canal wall [14]. This effect was fully described in a work by Mohn *et al*. [15] and appeared to happen immediately when ultrafine bioactive contents of up to 30 wt% had been incorporated into the gutta-percha matrix. Similarly, Resilon™ (Pentron^®^ Clinical Technologies, Wallingford, CT, USA) and its accompanying dual-cure methacrylic primer/sealer Epiphany™ (Pentron^®^ Clinical Technologies), also sold as RealSeal (SybronEndo, Orange, CA, USA), were introduced after fundamental research based on developing a bioactive obturation system that would fuse to dentine and form a monoblock within the canal [16]. According to Shanahan and colleagues [17], a strategic advantage was that, even in the absence of coronal restoration, leakage could be eliminated or dramatically reduced. The authors also suggested that this monoblock would be highly beneficial in order to strengthen the structure of the tooth attenuated by endodontic instrumentation. While this might be considered a secondary benefit as compared to its potential to ensure a thorough seal of the root canal system, it is not in any way inconsequential considering that gutta-percha does not reinforce weakened roots. To be used with any conventional obturation technique, Resilon™ points were developed in different tapers and ISO sizes from the synthetic polymer polycaprolactone, which is a thermoplastic aliphatic polyester, filled with radiopaque particles as well as B-G to impart bioactivity [17]. Use of a self-etch primer was advocated to improve adhesion of obturation core, sealer and dentine to one another, while simultaneously taking advantage of B-G’s unique characteristics: potential to displace water from the underfilled regions of the root canal system, redeposition of apatitic tooth mineral and formation of a physical bond with the moist dentine surface [7]. It was postulated that moisture would not eventually affect the initial micromechanical interlocking established by the hydrophilic primer/sealer components into open dentinal tubules, but actually help to drive crystallization reactions within and around the core material [14]. As this process can bind water over long periods of time and encourage reprecipitation of apatitic deposits, it was anticipated that it might contribute to limit bacterial leakage during long-term function [16]. Since its introduction, a number of reports have been published regarding various aspects of Resilon™ system which would offer an improvement over gutta-percha. Different studies provided preliminary evidence that when teeth were treated with Resilon™, the leakage behavior was an order of magnitude less than that found with conventional obturation materials and methods [17]. Healing rates for Resilon™-filled teeth in private practice Fig. (**1**) have been reported to be within the range of success rates for studies with treatment techniques mostly in university settings with gutta-percha root filling [18]. However, these preliminary results require more profound analysis, longer follow-up periods and a larger number of patients to tell if the inclusion of highly reactive silicate compounds, such as B-G, within the composition of root canal filling materials might prove an evidence-based replacement for gutta-percha which has an established research track record over many years.

## ENDODONTIC MICROSURGERY

The success rates of conventional endodontic therapy have been improving over the years, although persistence of pathologic periapical conditions is far from a rare condition [19]. This fact suggests a considerable need for further treatment. Endodontic microsurgery is indicated when an orthograde approach is not possible or has not yielded the desired healing outcome, and to secure a biopsy. The most common endodontic surgical procedure consists of periradicular curettage, root-end resection, preparation and filling with the help of an operating microscope. Different materials have been used to seal the canal system apically and prevent the egress of bacteria and their by-products into the surrounding tissues with varying results [20-22]. Surveys such as that conducted by Maltezos *et al.* [22] showed that an ideal retrofilling material should have the following properties: besides good adherence to the dentine, it should be easy to use clinically, biocompatible, and dimensionally stable over time. Clinically high, long-term success rates (84-92%) were reported for MTA^®^ cement (ProRoot; Dentsply Tulsa Dental Specialties, Tulsa, OK), a derivative of a type I ordinary Portland cement with 4:1 proportions of bismuth oxide added for radiopacity [23]. Likewise, clinical studies showed enhanced success over long periods with Super EBA™ (Bosworth Company, Skokie, IL), a zinc oxide-eugenol cement modified with ethoxybenzoic acid [24]. Several bone replacement materials were placed in the periradicular surgical cavities in the attempt to enhance bony healing. Reviewing the literature provided some studies that showed increased radiographic success consequent on grafting, particularly with large lesions (over 10 mm) [25]. Most materials, at best, elicit a neutral response when implanted into the human body. B-G however was seen to dissolve at a rate equal to that at which new host tissue is remodelled, serving as a biocompatible framework along which mesenchymal stem cells migrate due to its excellent osteoconductive ability [3]. As mentioned in the previous section, the mechanism of gradual dissolution of the glass matrix, concomitant with synthesis of new hard tissue on its surface, presupposes an initial exchange of ions and results in a build-up of HCA that is of the same composition as the normal mineral phase of bone [4]. B-G has also been reported to be osteoinductive, encouraging osteogenic precursors to proliferate and differentiate into matrix-producing osteoblasts Fig. (**2**). Entire classes of genes associated with osteoblast growth and differentiation, maintenance of extracellular matrix, as well as promotion of cell-cell and cell-matrix adhesion, are up-regulated by the dissolution products of B-G [26]. Moreover, the controlled release of soluble ionic species promotes recruitment of mucopolysaccharides and glycoproteins from adjacent tissues into an organic matrix rich of collagen fibers in intimate contact with newly formed crystals of HCA, facilitating bone regeneration uniformly throughout the defect. Readers should be aware that similar cellular events do not occur with any other material because of the lack of similar ionic stimuli [4]. *In vitro* studies have found that dissolution of ionic species also leads to an increase of the local pH at values between 11.4 and 11.8, which is strongly antibacterial [7] and thus particularly beneficial for dead space management of areas that are chronically infected. As often reported in the literature, B-G may have a barrier function that hinders faster-growing connective tissue from penetrating the bone cavity [27]. Since its introduction, the original B-G has been released as PerioGlas^®^ (now sold by NovaBone Products LLC, Alachua, FL, USA) demonstrating consistent results in a variety of bone regenerative treatments [3]. In periodontal surgical procedures, it has been used to stimulate bone regeneration, primarily in the treatment of interproximal bone defects [2]. This resulted in a 50-70% size reduction of bone cavities [28]. Radiographic evaluations conducted after osseous fill with PerioGlas ^®^ during endodontic surgery showed earlier bone regeneration Fig. (**3**) and a higher rate of success compared to merely sealing the apical part of the tooth [27]. Moreover, PerioGlas^®^ had a hemostatic effect after being applied to the spongiosa, as illustrated in other studies [3].

## CONCLUSION

The arena of dental materials science is continuing to evolve and, in fact, a new day has dawned. This new horizon is the increased use of silicate compounds, including B-G, either as filler materials/coatings for polymer structures or as synthetic bone graft substitutes to elicit specific biological responses. Information on treatment outcomes is essential for the decision-making process. We must adopt stricter controls and performance standards, particularly with the evaluation of new products and technologies, for generating robust data in clinical studies and reaching our goal to implement highest standards of endodontics. Thus, understanding the science, technology and properties of B-G is a very important need for the oral healthcare community. This is a field of intense research, which is clearly manifested in the increasing number of publications with regard to its clinical applications [2, 12]. A number of authors listed the many benefits of this material in endodontic therapy [7, 14, 15] and associated microsurgery [27]. B-G’s ingenious moisture-initiated setting reaction, concomitant with the notable *in situ* nucleation of HCA and its precursors to form a mineral matrix, as well as the ability to enhance osteoblast adhesion, revascularization and differentiation of mesenchymal stem cells, undoubtedly represents a major step forward on the path to ultimate endodontic success. Furthermore, the unique bacterial growth inhibiting feature of B-G granules gives distinct advantages in areas that are postoperatively prone to infection, contributing to the resolution of inflammatory responses and providing extraordinarily favourable conditions for an uneventful healing process. Present experience with B-G supports the convenience of this biomaterial for the elimination of osseous defects due to periradicular pathologies and surgeries over alternate forms of synthetic graft materials. The initial evaluation of biologically active silicate compounds as a possible addition to the root canal space obturation process has also shown its value; further studies as a part of head to head comparisons with traditional materials are necessary to confirm these preliminary findings.

## Figures and Tables

**Fig. (1):**
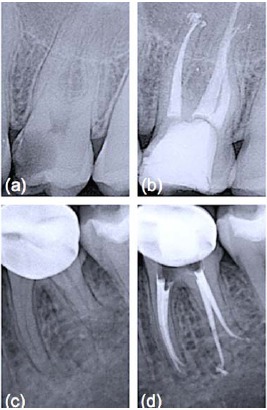
Periapical x-rays before and after root canal treatments. (a) Preoperative radiograph of upper left first molar (26); (b) One-year follow up radiograph of 26 obturated with vertically condensed gutta-percha and an epoxy-resin sealer; (c) Lower left first molar (36) before root canal treatment; (d) One-year follow up radiograph of 36 obturated with vertically condensed Resilon™ and RealSeal™ sealer. Note the similar radiopacity of the two filling materials (b and d).

**Fig. (2):**
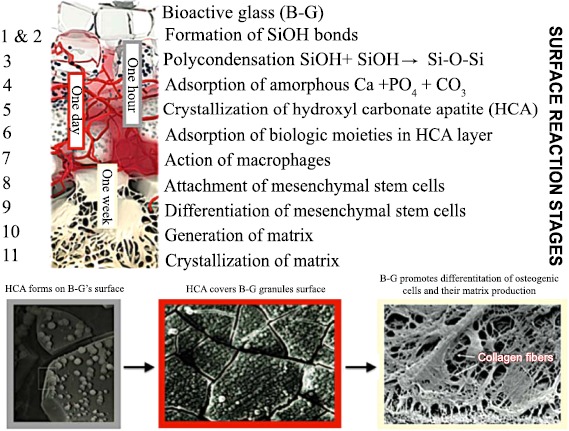
Timeline of molecular and cellular B-G reactions (modified after reference [4]). The process starts with five inorganic stages that occur very rapidly on the surface of B-G particles and lead to formation of polycrystalline HCA. The latter fastens down rapidly with the surrounding tissue from step 6 and acts as framework for the ingrowth of new bone. 3D architecture of mineralized bone is created by mesenchymal stem cells in response to critical concentrations of the soluble ionic constituents released from B-G. Mineralization of the matrix follows thereafter and mature osteocytes, encased in a collagen-HCA matrix, are the final product by 6-8 days *in vitro* and *in vivo*.

**Fig. (3):**
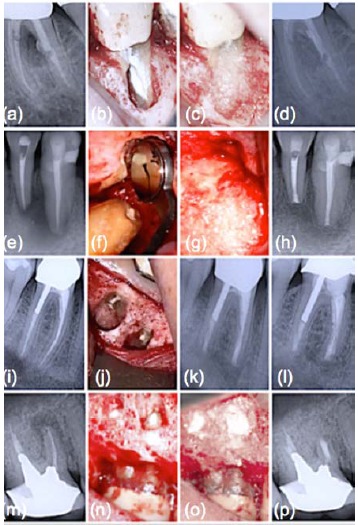
Clinical application of B-G during endodontic surgery. (a) Intraradicular post protruding through a perforation is noted radiographically; (b) Post reduction followed by root repair; (c) B-G placed into the surgical crypt prior to closure; (d) At one-year follow-up, resolution of furcal radiolucency; (e) Preoperative radiological situation; (f) Completed root-end filling; (g) B-G embedded in the bone cavity; (h) Resolution of radiolucency and reattachment of periodontal ligament at one-year control; (i) Preoperative periapical x-ray; (j) Retrofilling placed; (k) Immediate post-operative x-ray following apicoectomy with concomitant B-G grafting; (l) One-year follow up radiograph shows complete healing; (m) Preoperative periapical radiograph; (n) Terminated retrograde filling; (o) Intraoperative image showing B-G in place to fill in the bony cavity; (p) Radiological situation one year after surgery representing complete resolution of the periapical lesion.
